# Alterations in Glucose Metabolism During the Transition to Heart Failure: The Contribution of UCP-2

**DOI:** 10.3390/cells9030552

**Published:** 2020-02-27

**Authors:** Hanna Sarah Kutsche, Rolf Schreckenberg, Martin Weber, Christine Hirschhäuser, Susanne Rohrbach, Ling Li, Bernd Niemann, Rainer Schulz, Klaus-Dieter Schlüter

**Affiliations:** 1 Physiologisches Institut, Justus-Liebig-Universität, 35392 Gießen, Germany; Rolf.Schreckenberg@physiologie.med.uni-giessen.de (R.S.); bornemann-weber@t-online.de (M.W.); Christine.Hirschhaeuser@physiologie.med.uni-giessen.de (C.H.); Susanne.Rohrbach@physiologie.med.uni-giessen.de (S.R.); Ling.Li@physiologie.med.uni-giessen.de (L.L.); Rainer.Schulz@physiologie.med.uni-giessen.de (R.S.); Klaus-Dieter.Schlueter@physiologie.med.uni-giessen.de (K.-D.S.); 2 Universitätsklinikum Gießen, Klinik für Herz-, Kinderherz- und Gefäßchirurgie, 35392 Gießen, Germany; bernd.niemann@chiru.med.uni-giessen.de

**Keywords:** cardiomyocytes, cardiac remodeling, cardiac hypertrophy, uncoupling protein 2, glucose metabolism, heart failure

## Abstract

The cardiac expression of the mitochondrial uncoupling protein (UCP)-2 is increased in patients with heart failure. However, the underlying causes as well as the possible consequences of these alterations during the transition from hypertrophy to heart failure are still unclear. To investigate the role of UCP-2 mechanistically, expression of UCP-2 was silenced by small interfering RNA in adult rat ventricular cardiomyocytes. We demonstrate that a downregulation of UCP-2 by siRNA in cardiomyocytes preserves contractile function in the presence of angiotensin II. Furthermore, silencing of UCP-2 was associated with an upregulation of glucose transporter type (Glut)-4, increased glucose uptake, and reduced intracellular lactate levels, indicating improvement of the oxidative glucose metabolism. To study this adaptation in vivo, spontaneously hypertensive rats served as a model for cardiac hypertrophy due to pressure overload. During compensatory hypertrophy, we found low UCP-2 levels with an upregulation of Glut-4, while the decompensatory state with impaired function was associated with an increase of UCP-2 and reduced Glut-4 expression. By blocking the aldosterone receptor with spironolactone, both cardiac function as well as UCP-2 and Glut-4 expression levels of the compensated phase could be preserved. Furthermore, we were able to confirm this by left ventricular (LV) biopsies of patients with end-stage heart failure. The results of this study show that UCP-2 seems to impact the cardiac glucose metabolism during the transition from hypertrophy to failure by affecting glucose uptake through Glut-4. We suggest that the failing heart could benefit from low UCP-2 levels by improving the efficiency of glucose oxidation. For this reason, UCP-2 inhibition might be a promising therapeutic strategy to prevent the development of heart failure.

## 1. Introduction

Cardiac hypertrophy has been considered as an adaptive process that allows the heart to withstand systemic pressure overload. However, sustained hypertrophy may finally lead to heart failure. Therefore, a better understanding of the molecular processes that occur at the transition from hypertrophy to heart failure is essential to improve diagnosis and treatment options for heart failure patients [1].

Uncoupling proteins (UCPs) are a family of proteins located at the inner mitochondrial membrane [2]. UCP-1 was first identified in brown adipose tissue, where it uncouples the electron transport chain leading to heat production. In contrast, UCP-2 and UCP-3 are expressed in different tissues in mammalians. Whereas UCP-2 shows a constitutive mRNA expression in the heart, UCP-3 is mainly expressed in skeletal muscles. In contrast to mRNA expression, protein expression of UCP isoforms in the heart is less well documented [3].

We showed recently that in mice hearts, UCP-2 is mainly expressed in non-myocytes. Moreover, the absence of UCP-2 in mice improved cardiac adaptation to pulmonary arterial hypertension, mainly by increased fibrosis [4]. However, it remains elusive whether UCP-2 is also preferentially expressed in non-myocytes in other mammalians. Furthermore, a functional role for UCP-2 in cardiac tissue has not yet been clarified. Potential roles for UCP-2 in cardiomyocytes include mitochondrial uncoupling and subsequent reduction of mitochondrial-derived reactive oxygen species (ROS), but also substrate transport into mitochondria and finally alteration of glucose metabolism [5].

In contrast to the aforementioned missing data about cell-specific expression of UCP-2 in hearts and lack of clear identification of its function, an upregulation of UCP-2 mRNA under conditions of heart failure has been repeatedly reported [6,7]. This suggests a potential role for UCP-2 at the transition from cardiac adaptation to heart failure. In UCP-2 knockout mice, heart failure induced by acute pressure overload was attenuated and cardiac function preserved [8]. This finding suggests that the induction of UCP-2 contributes to the functional impairment in end-stage heart failure. However, the relevance of these findings for other models of heart failure and other species, as well as the identification of underlying mechanisms, remains elusive. Therefore, we studied in vitro and in vivo the expression of UCP-2 in rat cardiomyocytes and its effects on left ventricular (LV) remodeling in terms of functional and metabolic reactions during the transition to heart failure.

## 2. Materials and Methods

### 2.1. Animals and Ethical Concerns

All animal experiments conformed to the guidelines from Directive 2010/63/EU of the European Parliament on the protection of animals used for scientific purposes and were approved by authorities (permission numbers: GI 20/1 No. 76/2014 and 77/2014). Male Wistar rats, purchased from Janvier Labs (Saint-Berthevin, France), were used for in vitro analysis, while female spontaneously hypertensive rats (SHR), obtained from Envigo (Huntingdon, UK), were used as an in vivo model for LV hypertrophy. The aldosterone receptor antagonist spironolactone was administered by dietary intake (50 mg/kg/day, custom-made by Altromin GmbH, Lage, Germany). In addition, explanted ventricular tissues from the Cardiac Transplant Program in Halle/Saale as well as donor hearts were investigated in this study. Those hearts could not be transplanted for technical reasons, but none of them had evidence of an underlying cardiac disease judged by the surgeon or had suffered a cardiac trauma. Patient characteristics have been described previously [9]. The use of human tissue was approved by local ethics committees (65/10, 229/18) and conformed the principles outlined in the Declaration of Helsinki.

### 2.2. Isolation and Cultivation of Adult Rat Ventricular Cardiomyocytes

Ventricular cardiomyocytes and non-myocyte cell fraction of male Wistar rats (3–5 months old) were isolated as described previously [10,11]. Briefly, rats were sacrificed by cervical dislocation under deep anesthesia with isoflurane (5%). The hearts were excised and transferred into ice-cold saline solution. Subsequently, they were fixated on the cannula of a Langendorff system, followed by perfusion with Powell medium (NaCl 110 mM, KCl 2.5 mM, KH_2_PO_4_ 1.2 mM, MgSO_4_ × 7H_2_O 1.2 mM, Hepes 25 mM, d(+)-glucose-monohydrate 10 mM, pH adjusted to 7.4), containing collagenase (25 mg/50 mL, 265 U/mg, Worthington Biochemicals, Lakewood, CO, USA) and CaCl_2_ (25 µM) for 25 min at 37 °C. Ventricular tissue was minced and incubated in the abovementioned buffer for 5 min. The remaining cell solution was filtered through a nylon mesh (200 µm). After centrifugation and stepwise addition of CaCl_2_ (250, 500, and 1000 µM), cells were plated onto 35 mm culture dishes (Falcon, type 3001) coated with 4% (*vol*/*vol*) fetal calf serum (FCS). The supernatant was used as a non-myocyte cell fraction. Medium 199 was used as a cell culture medium supplemented by the following substances: carnitine (2 mM), creatine (5 mM), taurine (5 mM), penicillin-streptomycin (2%), and cytosine-β-arabinofuranoside (10 mM). The pH was adjusted to 7.4.

Myocytes used for the determination of cell shortening were cultured for 24 h in plain medium (see above). Load-free cell shortening was then analyzed using a cell-edge-detection system as initially described [12]. For long-term experiments, cells were cultivated for up to 5 days in the same medium with the addition of 20% fetal calf serum (FCS). Small interfering RNA (siRNA, 0.05 µM, QIAGEN, Venlo, The Netherlands) was used to decrease the expression of UCP-2 and Glut-4. Scrambled siRNA (scRNA, QIAGEN, Venlo, The Netherlands) served as a negative control. Genipin (10 µM dissolved in DMSO, Sigma-Aldrich, Taufkirchen, Germany) was used for pharmacological inhibition of UCP-2. To induce hypertrophy in cultured cardiomyocytes, cells were incubated with angiotensin II (Ang-II, Sigma-Aldrich, Taufkirchen, Germany) for either 24 h (0.01, 0.1, 1, and 10 µM) or for 5 days (1 µM).

### 2.3. Isolation of Mitochondria and Measurement of ROS and Respiration

Cardiac mitochondria were isolated from rats by differential centrifugation [13]. After scarification of the rats, hearts were rapidly excised into an ice-cold isolation buffer (50 mM sucrose, 200 mM mannitol, 5 mM KH_2_PO_4_, 1 mM EGTA, 5 mM MOPS, and 0.1% BSA, pH 7.15 adjusted with KOH), atria were removed, and 1 mm^3^ pieces of ventricular myocardium were homogenized (30 mL of isolation buffer per heart) using a PT10/35 Polytron (Brinkman Instruments, Westbury, NY, USA). Three 20 s homogenization cycles were performed on ice, and then the samples were centrifuged for 10 min (at 750× *g*) using a Sorvall II centrifuge equipped with a GSA rotor. The supernatant, containing the mitochondrial fraction, was further centrifuged at 7,000× *g* for 20 min, and the pellet was resuspended in 30 mL of isolation buffer (with no EGTA) and spun at 7,000× *g* for 20 min. Finally, mitochondria were resuspended in the isolation buffer (with no EGTA), and protein concentration was determined using a protein kit (Bio-Rad, Munich, Germany). Mitochondrial suspension (30–40 mg protein/mL) was kept on ice before experiments.

Respiration was analyzed as described before [14]. Briefly, oxygen consumption was measured with a Clark-type oxygen electrode (Oxygenmeter 782, Strathkelvin, Glasgow, UK) at 25 °C in incubation buffer (125 mM KCl, 10 mM Tris, 1.2 mM phosphate, 1.2 mM MgCl_2_ 0.02 mM EGTA, pH 7.4 (titrated with MOPS)). Respiration was analyzed in the presence of 5 mM glutamate and 2.5 mM malate. After recording basal oxygen consumption, respiration was stimulated by the addition of 40 µM ADP. Oxygen consumption was analyzed in nmol O_2_ × min^−1^ × mg protein^−1^.

Mitochondrial ROS generation was analyzed as described before [14]. Briefly, approximately 50 µg mitochondria were incubated with incubation buffer (see above) supplemented with 5 mM glutamate and 2.5 mM malate, 50 µM Amplex UltraRed (Thermo Fischer Scientific, Schwerte, Germany), and 0.1 U × ml^−1^ horseradish peroxidase. The fluorescence was measured continuously for 4 min with a Cary Eclipse spectrophotometer (Agilent Technologies, Santa Clara, CA, USA) at the excitation/emission wavelengths of 565/581 nm respectively. Background fluorescence of the buffer without mitochondria was subtracted and the slope (fluorescence in arbitrary units/time) was calculated.

### 2.4. Glucose Uptake

Isolated cardiomyocytes were cultivated for 48 h in FCS-containing media in the presence of siRNA directed against UCP-2 or scRNA, as indicated above. The culture medium was then changed into glucose- and serum-free medium. After 4 h of glucose-deprivation, cells were incubated with 2-deoxy-d-glucose (50 µM) and radioactively-labeled 2-deoxy-d-glucose (0.5 µl/mL, deoxy-D-glucose 2-[1-^14^C], Perkin Elmer, Waltham, MA, USA) for 90 min. Cells were washed thrice with phosphate buffered saline (PBS) and then dissolved in 1 M NaOH + 0.01% SDS overnight at 37 °C. The radioactivity was counted by a Tri-Carb 2810TR Low Activity Liquid Scintillation Analyzer (Perkin Elmer, Waltham, MA, USA) and was normalized to total protein concentration.

### 2.5. Intracellular Lactate Levels

Intracellular lactate concentrations were measured with a bioluminescent based assay according to the manufacturer’s protocols (Lactate-Glo™, Promega, Mannheim, Germany). Cardiomyocytes were cultivated for 48 h in FCS-containing media in the presence of siRNA directed against UCP-2 or scRNA. Cells were washed and then treated with an inactivation solution (0.6 N HCl) and neutralization solution (1 M Tris base) to inactivate the endogenous lactate dehydrogenase and to prevent NADH degradation. The cell lysates were then transferred into a 96-well plate and incubated with the assay reagent for 60 min. Luminescence signal was detected by a microplate reader (Infinite^®®^ M200, Tecan, Männedorf, Switzerland).

### 2.6. Cell Viability Assay

Cardiomyocytes were cultivated in FCS-containing media under control conditions or in the presence of either Ang-II, a combination of Ang-II and siUCP-2, or a combination of Ang-II, siUCP-2, and siGlut-4. An addition of 20% FCS enables long-term cultivation of primary cardiomyocytes, inducing a complex remodeling process, which was recently described [15]. To determine cell viability of cardiomyocytes after 5 days of cultivation, cells were double-stained with the DNA binding dyes Hoechst 33342 (1.2 mg/mL, Thermo Fisher Scientific, Schwerte, Germany) and propidium iodide (20 µg/mL, Sigma-Aldrich, Taufkirchen, Germany) and analyzed by fluorescence microscopy. Therefore, the fluorescent dyes were added to culture dishes and incubated for 5 min at 37 °C. Subsequently, five pictures of each culture dish with at least 150 cells per dish were randomly taken by a fluorescence microscope (Biozero BZ-8000K, Keyence, Neu-Isenburg, Germany). Hoechst 33342 is membrane permeable and stains DNA of vital and dead cells, visualized as a blue color by fluorescence microscopy. Since propidium iodide is membrane impermeable and can only enter cells with compromised membranes, necrotic cells are stained with red fluorescence. The percentage of viable cells was quantified blindly by counting the vital and the necrotic cells and calculated by the number of vital cells divided by the total cell number.

### 2.7. RNA Isolation and Real-Time PCR

Total RNA was extracted from isolated cells or tissues using peqGOLD TriFast (Peqlab, Biotechnologie GmbH, Erlangen, Germany), according to the manufacturer’s protocol. After conversion into complementary DNA (cDNA) with reverse transcriptase, PCR was performed using MyiQ™ detection systems (Bio-Rad, Munich, Germany) along with the iTaq Universal SYBR Green Real-Time PCR Supermix (Bio-Rad, Munich, Germany). A complete list of primers has been added to the Supplementary Material (Table S1). Quantification was performed as described before [16]. Data were normalized to hypoxanthine phosphoribosyl transferase (HPRT), as indicated.

### 2.8. Western Blots

Total protein was extracted from isolated cells or LV tissues using cell lysis buffer (Cell Signaling, Technology, Frankfurt, Germany), according to the manufacturer’s protocol. Briefly, the homogenates were centrifuged at 14,000× *g* for 10 min and the supernatants were treated with Laemmli buffer (Sigma-Aldrich, Taufkirchen, Germany). The protein concentration was adjusted to either 40 µg/µL for tissue extracts or 20 µg/µL for isolated cells. Recombinant hUCP-2 (kindly provided by Prof. Dr. E. Pohl, University of Veterinary Medicine, Vienna, Austria) was used as a positive control. Protein samples were loaded on NuPAGE Bis-Tris Precast gels (10%; Life Technology, Darmstadt, Germany) and subsequently transferred onto nitrocellulose membranes. The expression of UCP-2 was analyzed with an antibody (also kindly provided by Prof. Dr. E. Pohl), whose specificity was evaluated before [4,17]. The Glut-4 antibody was kindly provided by Samuel W. Cushman (NIH, National Institute of Diabetes and Digestive and Kidney Diseases, Bethesda, Montgomery, MD, USA). Expression of both proteins was normalized to the expression of glyceraldehyde 3-phosphate dehydrogenase (GAPDH) using an antibody produced in mice (Calbiochem^®^, Schwalbach, Germany). Secondary antibodies (horseradish peroxidase-coupled secondary antibody) directed against rabbit IgG or mouse IgG were purchased from Dako (now Agilent Technologies, Santa Clara, CA, USA).

### 2.9. Assessment of LV Hypertrophy and Function

The systolic and diastolic blood pressure and the heart rate of SHR were measured using non-invasive tail-cuff blood pressure measurement (209000 Series, TSE-Systems, Bad Homburg, Germany). Prior to the start of the experiment, the animals were adjusted to the experimental procedure over a week. The median of 10 consecutive measurements was calculated for each parameter described. For echocardiographic analysis, rats were anesthetized by isoflurane inhalation (2%; 98% O_2_). LV function was assessed by two-dimensional echocardiography using a 12.5 MHz probe (Vivid I, GE Health Care, Chicago, IL, USA). All measurements were in accordance with the conventions of the American Society of Echocardiography and conducted by the same trained, blinded sonographer, as described before [18].

### 2.10. Statistics

Data were analyzed for normal distribution (Shapiro–Wilk test) and variance (Levene test). Subsequently, data were analyzed by *t*-test, Welch’s *t*-test or the Mann–Whitney test (two samples) or ANOVA with a Student–Newman–Keuls post hoc analysis (more than two groups). If possible, exact *p*-values are given for all analysis with *p* < 0.05. SPSS 22.0 was used to calculate the data.

## 3. Results

### 3.1. Effect of UCP-2 Expression and Activity on Load-Free Cell Shortening of Adult Rat Ventricular Cardiomyocytes

**Figure 1:**
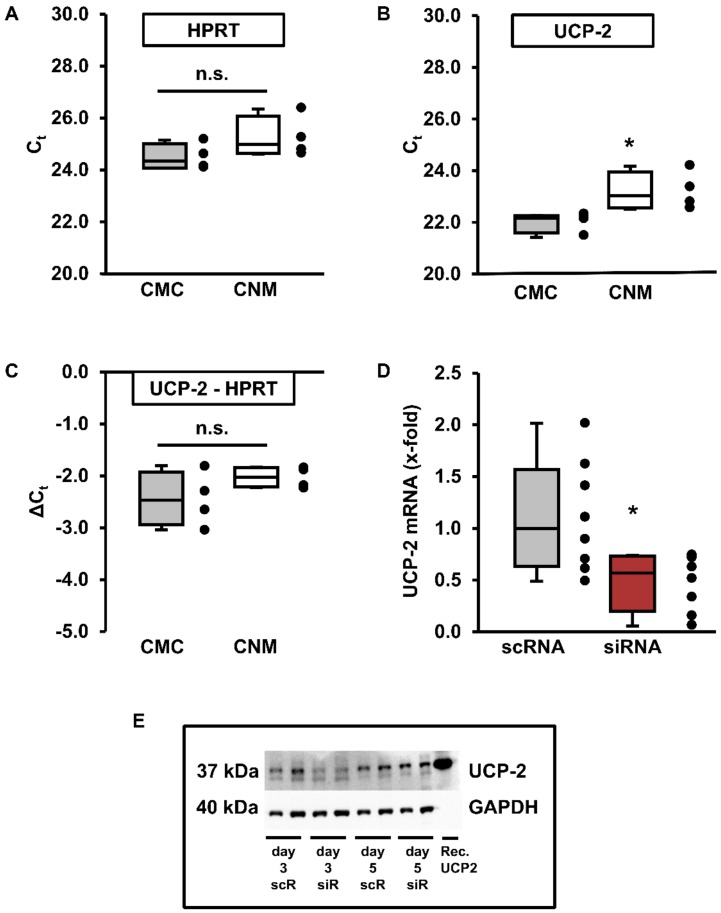
Basal expression levels of UCP-2 in adult rat ventricular cardiomyocytes and cardiac non-myocytes and UCP-2 expression in cardiomyocytes after siRNA treatment. (**A–B**) Real-time RT–PCR cycle thresholds (C_t_) for the housekeeping gene HPRT (**A**), UCP-2 (**B**) and UCP-2 after normalization to HPRT (C_t_) expression (**C**) in cardiomyocytes (CMC) and cardiac non-myocytes (CNM). Data are from *n* = 4 preparations and statistically analyzed using unpaired *t*-test (* *p* = 0.02959). (**D**) Relative UCP-2 mRNA expression in cells exposed to small inhibitory (siRNA) against UCP-2 or scrambled RNA (scRNA) for 24 h. Data are from *n* = 8 cultures out of 4 individual preparations and statistically analyzed using unpaired *t*-test (* *p* = 0.011). (**A**–**D**) Data expressed as 25%, 50%, and 75% quartiles with whiskers representing the total range, and dots representing the individual data points. (**E**) Original Western blot showing expression of UCP-2 in cardiomyocytes after 3 or 5 days in the presence of scRNA or siRNA directed against UCP-2. Rec. UCP-2 = control peptide.

At first, we analyzed basal gene expression of UCP-2 in terminal differentiated rat cardiomyocytes and compared its expression to that of non-myocytes. HPRT was used as a house-keeping gene to normalize to equal loading as it showed comparable expression in myocytes and non-myocytes (Figure 1A). UCP-2 expression was slightly higher in cardiomyocytes than in non-myocytes (*p* < 0.05; Figure 1B). However, after normalization to the house-keeping gene, UCP-2 expression was not different (Figure 1C). Nevertheless, in contrast to mice hearts, cardiac myocytes and non-myocytes from rat hearts showed comparable expression of UCP-2 mRNA. In order to analyze the effects of UCP-2 on cellular function, it was necessary to reduce its expression in cardiomyocytes. Administration of siRNA to adult rat ventricular cardiomyocytes reduced the corresponding UCP-2 mRNA expression by more than 40% (Figure 1D). Similarly, UCP-2 protein levels were also diminished after three days of cultivation (Figure 1E).

**Figure 2:**
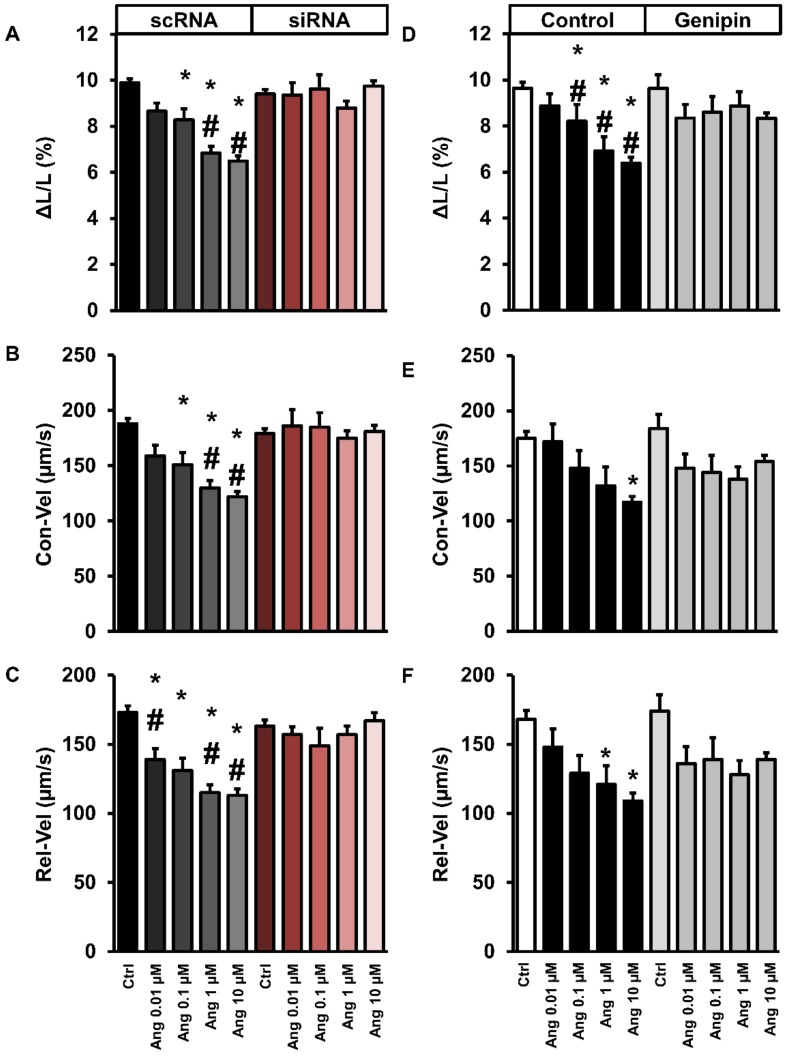
Effects of UCP-2-silencing or genipin treatment on cell shortening (**A**,**D**), contraction velocity (**B**,**E**), and relaxation velocity (**C**,**F**) after hypertrophic stimulation. (**A–C**) Cardiomyocytes were exposed to scRNA or siRNA and incubated with angiotensin-II (Ang-II 0.1–10 µM) or vehicle (Ctrl). Data are means ± SD. from 189–252 cells (Ctrl), 36 cells (Ang-II 0.01 and 0.1 µM), 144 cells (1 µM) or 153–198 cells (10 µM). (**D–F**) Cardiomyocytes were exposed to genipin (10 µM) or vehicle (control with DMSO) and incubated with Ang II (0.1–10 µM) or vehicle. Data are means ± S.D. from *n* = 108 (Ctrl), 36 (Ang-II 0.01 and 0.1 µM), 126 (Ang-II 1 and 10 µM). A one-way ANOVA with a subsequent Student–Newman–Keuls test for post hoc analysis was performed (* *p* < 0.05 vs. Ang-II-free; ^#^ *p* < 0.05 vs. siRNA- or genipin-treated cells).

The renin-angiotensin-aldosterone-system (RAAS) plays a pivotal role in the progression of heart failure. This process can be monitored by reduced load-free cell shortening of isolated adult rat ventricular cardiomyocytes. After incubation with Ang-II, load-free cell shortening (quantified by percent cell shortening (ΔL/L), contraction velocity, and relaxation velocity) was decreased in a concentration-dependent way (Figure 2A–C). However, in cells exposed to siRNA directed against UCP-2, these effects were completely attenuated (Figure 2A–C). Similarly, a reduction of UCP-2 activity by pharmacological inhibition using genipin was sufficient to attenuate these effects again (Figure 2D–F).

### 3.2. Effects of UCP-2 Expression and Activity on Mitochondrial Function, Metabolism, and Viability

**Figure 3:**
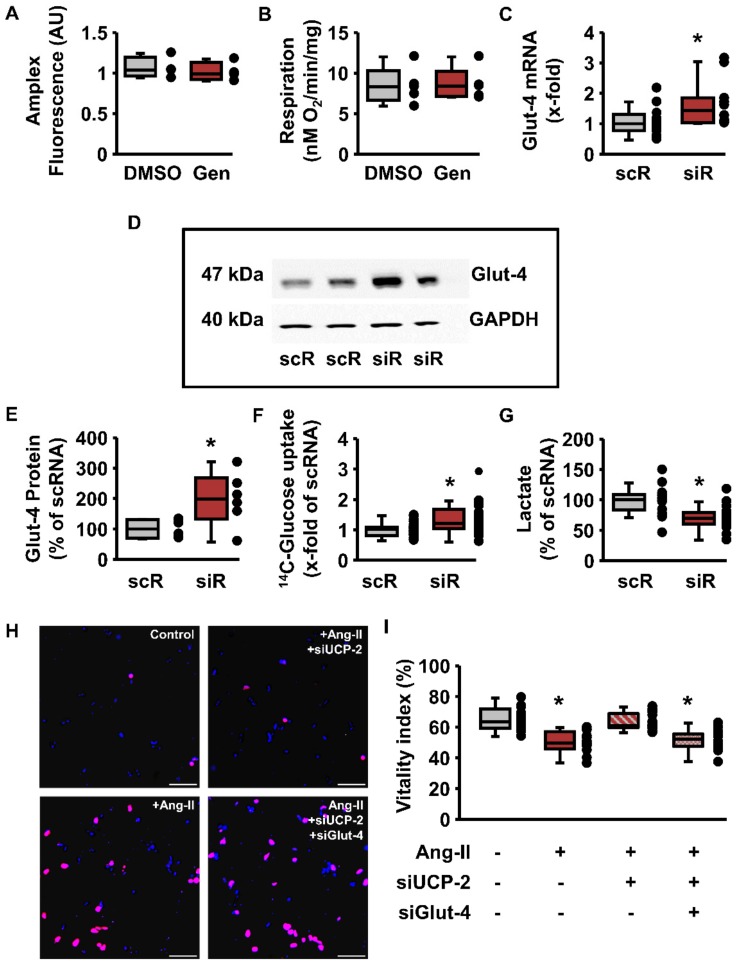
Effect of UCP-2 silencing or inhibition on mitochondrial function, glucose metabolism, and viability upon Ang-II stimulation. (**A–B**) LV mitochondria were isolated and ROS formation (**A**) or respiration (**B**) was determined in the absence (control with DMSO) or presence of genipin (Gen, 10 µM) from *n* = 4 (**A**) or *n* = 5 (**B**) hearts. Statistical analysis was performed with unpaired *t*-test (*p* > 0.05). (**C**) mRNA expression of Glut-4 in cells exposed to siRNA directed against UCP-2 or scrambled RNA (scRNA). Statistical analysis was performed with a Mann–Whitney test, from *n* = 12 cultures out of 6 individual preparations (* *p* = 0.038). (**D**) Original Western blot showing expression of Glut-4 in cardiomyocytes exposed to siRNA against UCP-2 (siR) or scRNA (scR). (**E**) Quantification of Western blots as shown in (**D**) normalized to GAPDH and compared to the basal expression in cultures exposed to scRNA. Data from *n* = 6 cultures out of 3 individual preparations. Statistical analysis was performed with unpaired *t*-test (* *p* = 0.030). (**F**) Glucose uptake of cardiomyocytes exposed to siRNA directed against UCP-2, normalized to the controls. Data are from *n* = 29 cultures out of 6 individual preparations and statistically analyzed using Welch’s *t*-test (* *p* = 0.001). (G) Intracellular lactate concentration in cardiomyocytes exposed to siRNA directed against UCP-2, normalized to the mean of the controls (scRNA) with *n* = 19 cultures out of 3 individual preparations. Statistical analysis was performed with unpaired *t*-test (* *p* = 0.00026). (**H–I**) Viability assay by Hoechst 33342 and propidium iodide staining of isolated cardiomyocytes. Cells were treated with Ang-II (1 µM) alone, to induce hypertrophic remodeling or in the presence of Ang-II and Glut-4 siRNA and/or UCP-2 siRNA, respectively. (**H**) Representative images of necrotic (propidium iodide positive, red) and vital (Hoechst 33342 positive, blue) cells after 5 days of cultivation in the presence of 20% FCS. The different treatment groups are indicated. Scale bars: 200 µM. (**I**) Proportion of vital cells (stained positive for Hoechst 33342 and negative for propidium iodide), from *n* = 16 cultures out of 4 individual preparations. A one-way ANOVA with a subsequent Student–Newman–Keuls test for post hoc analysis was performed (* *p* < 0.05 vs. Ang-II-free and vs. Ang-II + siUCP-2). Data expressed as 25%, 50%, and 75% quartiles, with whiskers representing the total range and dots representing the individual data points.

Although an exact role of UCP-2 in cardiomyocytes is still unknown, it has been suggested that high expression of UCP-2 reduces mitochondrial ROS formation. However, inhibition of UCP-2 activity by genipin did not affect ROS production in isolated mitochondria from rat hearts (Figure 3A). Furthermore, inhibition of UCP-2 by genipin did not affect mitochondrial respiration (Figure 3B).

As it was proposed that UCP-2 affects glucose metabolism [19,20,21], the effect of UCP-2 silencing on the expression of glucose transporters was investigated next. In fact, a reduction of UCP-2 was associated with an upregulation of Glut-4 on both the mRNA and protein levels (Figure 3C–E). Moreover, in cells treated with siRNA directed against UCP-2, glucose uptake was significantly increased (Figure 3F). To determine whether UCP-2 downregulation enhances mitochondrial glucose oxidation, intracellular lactate accumulation was measured. UCP-2 silencing led to significantly decreased lactate levels (Figure 3G). To study the effects of UCP-2 and Glut-4 silencing on the viability of cardiomyocytes under stress conditions, cells were incubated with Ang-II for five days to induce adverse remodeling. Incubation with Ang-II significantly reduced cardiomyocyte vitality, which was prevented by the addition of UCP-2 siRNA (Figure 3H,I). In contrast, combining UCP-2- and Glut-4 silencing, Ang-II led to the same reduction in cardiomyocyte vitality as found under stimulation with Ang-II alone (Figure 3H,I).

### 3.3. Regulation of UCP-2 and Glut-4 During Chronic Hypertension

**Figure 4:**
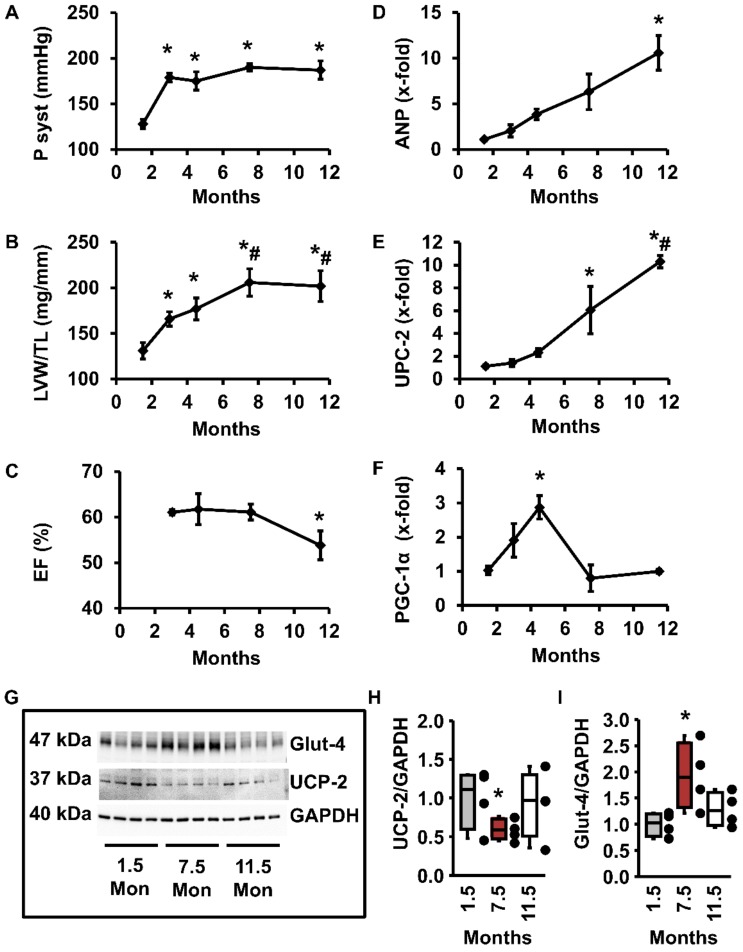
Blood pressure and hypertrophy in spontaneously hypertensive rats (SHR). (**A**) Systolic blood pressure in SHRs at various time-points (1.5 months *n* = 4; 11.5 months *n* = 9; all others *n* = 6). * *p* < 0.05 vs. 1.5 months. (**B**) LV weight normalized to tibia length (LVW/TL; 1.5 months: *n* = 8; 11.5 months: *n* = 5; all others *n* = 10). * *p* < 0.05 vs. 1.5 months; ^#^ *p* < 0.05 vs. 1.5–4.5 months. (**C**) Ejection fraction (EF) in SHR (*n* = 5–6). * *p* < 0.05 vs. 3 months. ANP (**D**), UCP-2 (**E**) and PGC-1α (**F**) mRNA expression in the LV of SHRs (1.5–7.5 months *n* = 6; 11.5 months *n* = 12; * *p* < 0.05 vs. 1.5 months; ^#^ *p* < 0.05 vs. 1.5–7.5 months). (**G**) Original Western blot showing Glut-4, UCP-2, and GAPDH expression in the LV of SHRs at an age of 1.5, 7.5, and 11.5 months. (**H**,**I**) Quantification of protein levels from Western blots of *n* = 4 independent experiments, normalized to GAPDH (*p* < 0.05 vs. 1.5 and 11.5 months). (**A**–**F**,**H**,**I**) Data expressed as 25%, 50%, and 75% quartiles, with whiskers representing the total range and dots representing the individual data points. Statistical analysis was performed with a one-way ANOVA with a subsequent Student–Newman–Keuls test for post hoc analysis.

The aforementioned results on isolated adult rat ventricular cardiomyocytes suggest that UCP-2 downregulation is associated with increased glucose uptake. A metabolic shift from the preferential use of fatty acids to carbohydrates is part of the hypertrophic adaptation of the heart to pressure overload. The inverse regulation of UCP-2 and Glut-4 may be involved in this mechanism. Therefore, we analyzed the time-dependent regulation of UCP-2 und Glut-4 in a model of essential hypertension (spontaneously hypertensive rats, SHR). Up to the 8th week of life, SHR have normotensive blood pressure values of 125/80 mmHg. Then they develop arterial hypertension over a period of 2 months, with values of 175/115 mmHg (Figure 4A). The steady rise in blood pressure is followed by severe LV hypertrophy (Figure 4B). At the latest observation point (12 months of age), cardiac function declined as indicated by reduced LV ejection fraction (Figure 4C). Cardiac hypertrophy was accompanied by an increase in the expression of atrial natriuretic peptide (ANP), an established marker of ventricular hypertrophy (Figure 4D). Similarly, UCP-2 mRNA expression increased excessively at the age of 12 months—that is, when ejection fraction significantly declined (Figure 4E). Interestingly, the mRNA expression of PGC-1α, associated with mitochondrial formation increased in the early, adaptive phase of cardiac hypertrophy, but declined thereafter, when the expression of UCP-2 was induced and function worsened (Figure 4F). More importantly, UCP-2 protein expression showed a biphasic expression during our observation period: after an initial decline in UCP-2 protein expression during the onset of hypertrophy, UCP-2 expression rose significantly at later time-points (Figure 4G–I). As expected from our in vitro studies (see above), Glut-4 protein expression was regulated to the contrary: it rose initially, when UCP-2 expression was low, and declined at later time-points when UCP-2 expression increased again (Figure 4G–I).

### 3.4. Effect of Spironolactone on the Expression of UCP-2 and Glut-4 in Pressure-Overloaded Hearts

Results from our in vitro and in vivo experiments suggest that upregulation of UCP-2 and a corresponding down-regulation of Glut-4 participate in the progression of heart failure under conditions of chronic pressure overload. If this hypothesis is correct, a treatment strategy to avoid the re-induction of UCP-2 at that time should prevent such a transition. Aldosterone treatment in rats was shown to impair glucose uptake in the liver and skeletal muscle via inhibition of Glut-2 and Glut-4 gene expression and decrease translocation of Glut-4 to the plasma membrane [22]. Spironolactone, an aldosterone blocker (mineralocorticoid receptor antagonist, MRA), was shown to improve insulin sensitivity and glucose transport [23]. More importantly, spironolactone was able to prevent a chlorthalidone-induced insulin resistance in hypertensive patients, which was not observed when chlorthalidone was combined with the Ang-II receptor blocker irbesartan [24]. Thus, we aimed to determine if blocking the aldosterone receptor during hypertrophic remodeling prevents the decline of Glut-4 expression by reducing UCP-2 expression. Therefore, we started to treat SHRs at the age of 6 months (with established myocardial hypertrophy but preserved function) for the subsequent next four months with spironolactone.

**Figure 5:**
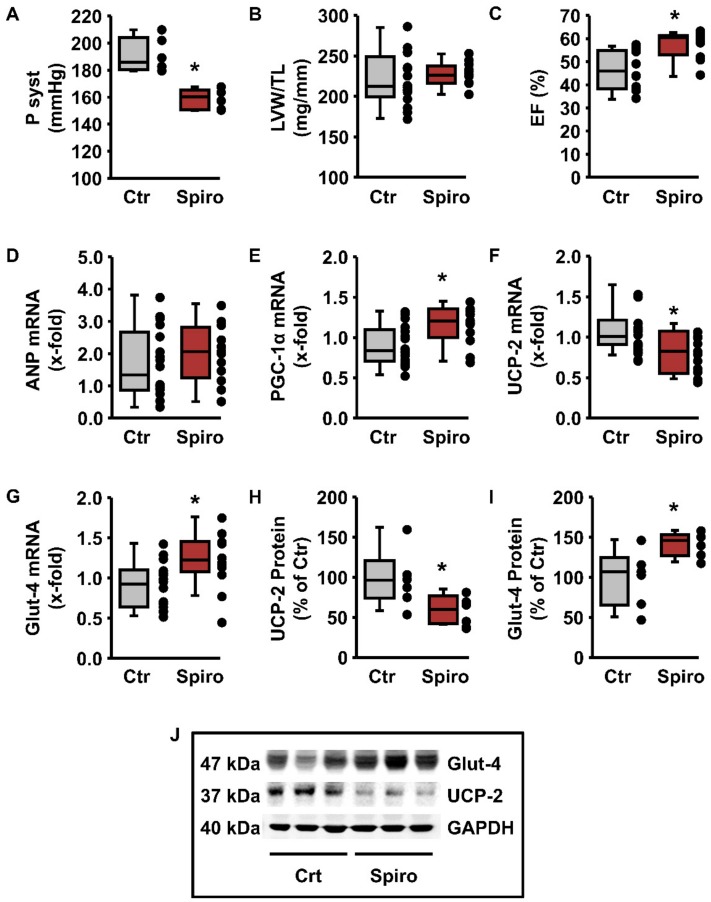
Blood pressure and hypertrophy in 10-months old spontaneously hypertensive rats (SHR) treated with (Spiro) or without (Ctr) spironolactone for the last four months. (**A**) Systolic blood pressure in SHRs (*n* = 6). Statistical analysis was performed with unpaired *t*-test (* *p* = 0.00035). (**B**) LV weight normalized to tibia length (LVW/TL; *n* = 12–16). Statistical analysis was performed with Welch’s *t*-test (*p* > 0.05). (**C**) Ejection fraction (EF) in SHR (*n* = 10–12). Statistical analysis was performed with a Mann–Whitney test (*p* = 0.00193). (**D–G**) mRNA expression of ANP (**D**), PGC-1α (**E**), UCP-2 (**F**) and Glut-4 (**G**) in the LV of SHRs (*n* = 12–18). Statistical analysis was performed with unpaired *t*-test (*p* > 0.05 (ANP); * *p* = 0.00664 (PGC-1α); * *p* = 0.00704 (UCP-2); * *p* = 0.01404 (Glut-4)). (**H, I**) Quantification of protein levels from Western blots showing Glut-4, UCP-2, and GAPDH expression in the LV of SHRs at an age of 11.5 months, normalized to GAPDH and compared to the basal expression of the controls. Data are from *n* = 6 independent experiments and are statistically analyzed using unpaired *t*-tests. (**H**) UCP-2 (* *p* = 0.026) and (**I**) Glut-4 (* *p* = 0.015). (**J**) Original Western blot showing Glut-4, UCP-2, and GAPDH expression in the LV of SHRs. (**A**–**I**) Data are expressed as 25%, 50%, and 75% quartiles, with whiskers representing the total range, while the dots represent the individual data points.

Treatment with spironolactone slightly reduced blood pressure (Figure 5A), did not affect hypertrophy (Figure 5B), did not affect the mRNA expression of ANP (Figure 5D) but attenuated the fall in ejection fraction (Figure 5C). However, it increased the expression of PGC-1α (Figure 5E) while it subsequently reduced the expression of UCP-2, and increased that of Glut-4 (Figure 5F–G). This was also confirmed on the protein level (Figure 5H–J). Consequently, starting MRA therapy during the stage of compensated hypertrophy should prevent the downregulation of Glut-4 and thereby stabilize cardiac metabolism and function.

### 3.5. UCP-2 and Glut-4 in Human End-Stage Heart Failure

**Figure 6:**
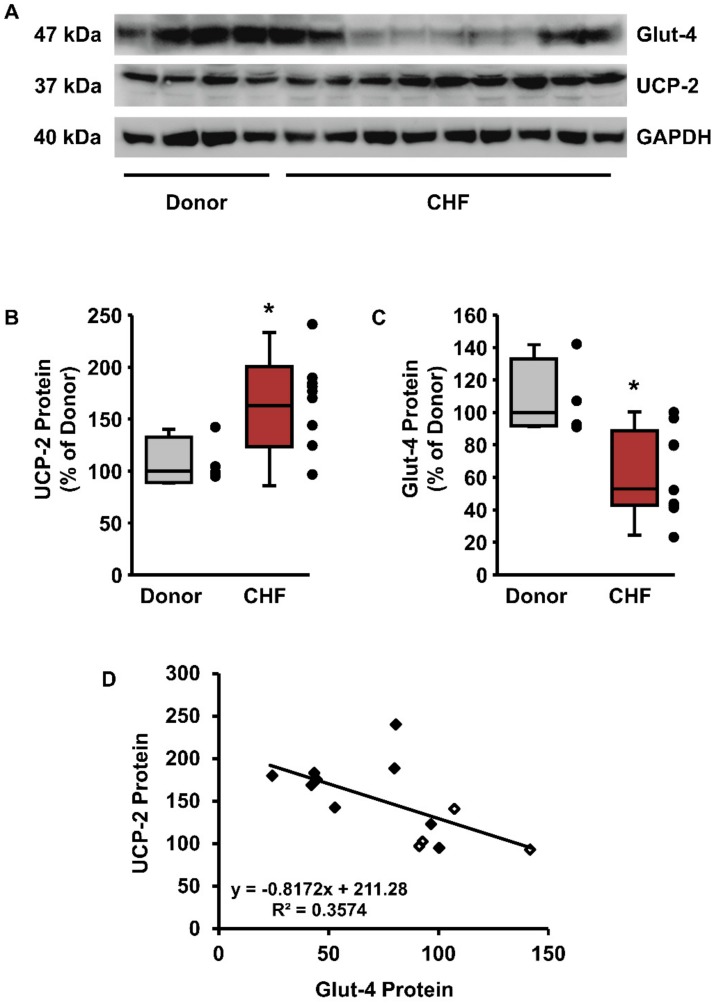
UCP-2 and Glut-4 expression in LV from healthy donor hearts or explanted hearts of end-stage congestive heart failure (CHF). (**A**) Original Western blot. (**B**,**C**) Quantification of protein levels from Western blots normalized to GAPDH and compared to the expression of donor hearts. Data from *n* = 4 (Donor) or *n* = 9 (CHF) hearts are expressed as 25%, 50%, and 75% quartiles, with whiskers representing the total range. The dots represent the individual data points. Statistical analysis with unpaired *t*-test. (**B**) UCP-2 (* *p* = 0.026) and (**C**) Glut-4 (* *p* = 0.015). (**D**) Association between UCP-2 and Glut-4 expression with Pearson’s correlation coefficient (*p* = 0.03093). Open symbols: donor hearts. Filled symbols: CHF.

Finally, we addressed the question of whether a similar effect of UCP-2 and Glut-4 expression holds in human hearts at end-stage heart failure. Protein levels of LV from patients with end-stage heart failure (transplanted hearts) displayed a higher expression of UCP-2 compared to donor hearts, but a reduced expression of Glut-4 (Figure 6A–C). Moreover, we found a reverse correlation between UCP-2 and Glut-4 expression by pair-to-pair comparison of the expression in individual patients (Figure 6D).

## 4. Discussion

**Figure 7:**
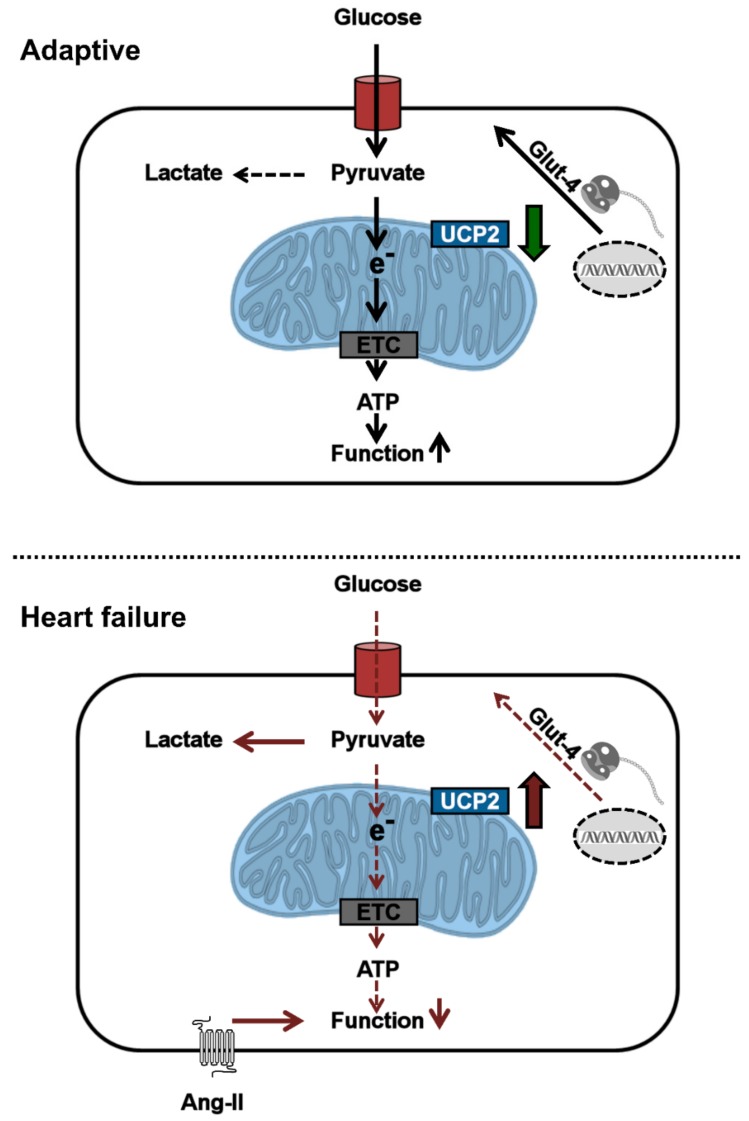
Final summary. During the adaptive phase of myocardial hypertrophy UCP-2 expression is reduced and Glut-4 expression is increased. We hypothesize that this improves pyruvate import into mitochondria and maintains cardiomyocyte function. During the transition to heart failure (HF), UCP-2 expression is increased and, subsequently, Glut-4 expression reduced. UCP-2 expression would then inhibit pyruvate entry and glucose would be metabolized in a glycolytic pathway leading to decreased ATP levels and lactate accumulation. e- = electron. ETC = electron transport chain.

The current study aims to clarify the role of UCP-2 during the transition from cardiac hypertrophy to heart failure. The most important findings of our study are (1) that silencing of UCP-2 in cultured adult rat ventricular cardiomyocytes improves glucose uptake and preserves cell function in an Ang II-driven model of cell dysfunction, (2) that UCP-2 expression is transiently down- and glucose uptake clearly up-regulated during the adaptive phase of cardiac hypertrophy, but (3) that an up-regulation of UCP-2 at the time of decompensation is associated with less Glut-4 expression and reduced function. Finally, we were able to show that these mal-adaptive processes during the late stage of hypertrophy can be attenuated by blockade of aldosterone pathways. Figure 7 summarizes these findings.

In a previous study, we found that UCP-2 expression in cardiomyocytes from mice is low compared to non-myocytes and that a deficiency of UCP-2 is accompanied by improved fibrotic responsiveness in the right ventricle, which preserves cardiac output under pressure overload [4]. However, the situation in a pressure-overloaded LV may be different as fibrosis contributes to the development of LV diastolic dysfunction [18]. Moreover, recent studies suggest differences in the cardiac expression of UCP-2 between mice and rats, indicating a higher expression of UCP-2 in rat hearts [25]. UCP-2 seems to be phylogenetically selected for its role in cardiac tissue from rats. Therefore, we analyzed the expression and function of UCP-2 in adult rat ventricular cardiomyocytes.

We have not only found a constitutively high expression of UCP-2 in rat hearts but also a similar expression level between cardiomyocytes and non-myocytes, which represents another difference between mice and rats and suggests a specific role for UCP-2 in rat hearts. Our data contradict previous findings suggesting that mice and rat hearts do not express UCP-2 [26].

However, there are serious concerns about the ability of different antibodies to properly detect the level of UCP-2 protein. The antibody used in this study was validated using protein lysates from wild-type and UCP-2 knockout mice, as described before [4,17].

Having successfully established an in vitro model to study the effects of UCP-2 in isolated heart muscle cells by small interfering RNA, we proceeded to identify potential mechanisms by which UCP-2 may affect cardiac hypertrophy and failure. In general, high levels of UCP-2 should reduce ROS formation in the heart by lowering the mitochondrial membrane potential and therefore must be considered as a protective measure [27,28]. Additionally, UCP-2 was found to be activated by ROS [29]. Reduction of UCP-2 expression in adult rat ventricular cardiomyocytes by hyperglycemia increases ROS formation and induces cardiomyocyte contractile dysfunction [30]. However, whether UCP-2 affects electron transport chain activity is highly questionable. In contrast to this proposed mechanism, it was demonstrated that in mouse heart UCP-2 revealed beneficial effects on mitochondria, but without affecting ROS production [31]. Even more contradictory is the finding that in neonatal cardiomyocytes, ROS triggers the downregulation of UCP-2 and, thereby, accelerates the development of hypertrophy [32]. Moreover, we did not find any differences in mitochondrial function in the hearts of UCP-2-deficient mice compared with those of wild-type mice [4]. Similarly, in this study, we did not find any differences in ROS production in cardiac rat mitochondria treated with genipin, an inhibitor of UCP-2. These data contradict the assumption that UCP-2 acts as a modifier of electron transport chain activity in rat cardiomyocytes.

In contrast to the ROS hypothesis, other researchers favor a more recent approach for UCP-2 as a key regulator of metabolic pathways. For example, high UCP-2 levels may favor the use of free fatty acid oxidation in cardiac mitochondria [19,31]. Vice versa, free fatty acids have been reported to induce the expression of UCP-2 [33,34]. During the development of cardiac hypertrophy, the situation may differ: under these conditions, a metabolic shift from fatty acid consumption to glucose consumption is observed. This would then require a downregulation of UCP-2 and upregulation of glucose transporters. Glut-1 and Glut-4 are the most abundant glucose transporters in the heart [35]. Specifically, for the insulin-sensitive Glut-4, an inverse regulation between UCP-2 and Glut-4 was reported several times in skeletal muscles that were impaired by diabetes [36,37]. In the pancreas and in adipocytes, antidiabetic medication induces Glut-4 and reduces UCP-2 expression [38,39,40,41]. In the heart, an induction of UCP-2 was associated with reduced expression of Glut-4 [42,43]. Using the isolated cell culture model, we confirmed such a relationship for the first time in isolated cardiomyocytes. Silencing of UCP-2 was associated with an increased mRNA and protein expression of Glut-4 and increased glucose uptake.

In quiescent human pluripotent stem cells (hPSC), UCP-2 was found to regulate energy metabolism by preventing mitochondrial glucose oxidation [44]. Furthermore, during hPSC differentiation, the repression of UCP-2 seems to be necessary to switch from glycolysis to mitochondrial glucose oxidation, as ectopic UCP-2 expression disturbed this transition and impaired hPSC differentiation. Moreover, it was published that UCP-2 silencing in human hepatocellular carcinoma (HepG2) cells decreases lactate production and increases the ATP:ADP ratio, indicating that UCP-2 favors glycolytic pathways [21]. Within this study, it has been shown that UCP-2 transports C4 metabolites from mitochondria to the cytosol. Therefore, it was assumed that UCP-2 limits mitochondrial oxidation of pyruvate and enhances glycolysis and glutaminolysis. Also, in primary murine embryonic fibroblasts, it was shown that loss of function of UCP-2 increases proliferation, associated with a metabolic switch from fatty acid oxidation to glucose metabolism [20]. Additionally, ROS production was not increased. In line with these results, our data indicate that the downregulation of UCP-2 favors oxidative pathways, as assumed by decreased lactate accumulation. As cultured cells prefer glucose substrate utilization, an increase in oxidative phosphorylation of glucose by UCP-2 would be beneficial, by providing the cells with enough energy and limiting lactic acidosis. In accordance with this is the observation that UCP-2 over-expression in isolated rat cardiomyocytes led to a significant decline in ATP level and the development of acidosis [45]. More importantly, when cardiomyocytes were challenged with hypoxia-reoxygenation, a situation where cells switch to glycolytic energy generation, UCP-2 silencing prevented cell death [45].

To investigate these mechanisms under pathological situations, adverse remodeling of cardiomyocytes was induced by Ang-II. Activation of the RAAS is a key step in the progression of heart failure. In SHRs, a RAAS-driven model of essential hypertension, an upregulation of cardiac UCP-2 expression has already been described [46]. In isolated cardiomyocytes Ang-II induced the expression of UCP-2 and accelerated cellular aging [47]. We have previously shown that Ang II induces a decline of load-free cell shortening of isolated cardiomyocytes that mimics the situation in the whole heart as well [48]. In conclusion, the available data indicates that Ang-II induces the expression of UCP-2 and a corresponding decline in cardiac function. Given the importance of these cellular and molecular mechanisms which may directly contribute to the development of heart failure, we decided to investigate these findings in more detail. The results of our study show that the silencing of UCP-2, as well as inhibition of UCP-2 activity, are sufficient to abrogate the detrimental effect of Ang-II on isolated cardiomyocytes. This protective effect of UCP-2 silencing was abolished by the simultaneous inhibition of Glut-4 expression. Therefore, these protective effects seem to be driven by metabolic transitions through UCP-2, improving cardiomyocyte’s energy production.

Having established a potential mechanism by which UCP-2 may influence the remodeling of cardiomyocytes in vitro, it was important to translate these results into an in vivo setting by using SHRs. These rats display an induction of myocardial hypertrophy (adaptive) during the first six months of hypertension with a mal-adaptive remodeling in the following months. Results from these experiments show that, initially, UCP-2 is downregulated, together with an increased expression of PGC-1α and Glut-4. Mechanistically, it has been shown that Sirt-1 is required for UCP-2 downregulation by forming a complex with FoxO3a/PGC-1α [49]. In the second (mal-adaptive) phase, UCP-2 expression rises again with a subsequent downregulation of Glut-4. During this time, PGC-1α expression declines, indicating a decline in mitochondrial density. In a hypertrophic heart that has lost its metabolic flexibility due to the preferential substrate utilization of glucose, the described mechanisms may contribute to or accelerate the development of heart failure.

Following this line, SHRs were treated with spironolactone starting at an age of six months, before the transition from adaptive to mal-adaptive hypertrophy occurs. Indeed, cardiac function was maintained, UCP-2 expression remained low, and Glut-4 expression remained high. These data indicate that aldosterone might be responsible for the upregulation of UCP-2.

As mentioned before, major differences occur between rats and mice regarding the expression and cellular distribution of UCP-2 in the heart. Therefore, it is important to analyze whether the aforementioned regulation of UCP-2 and Glut-4 is also observable in the human heart. By comparing the expression of UCP-2 and Glut-4 between healthy donor hearts and hearts from patients after heart transplantation (end-stage heart failure), we observed a similar inverse correlation between UCP-2 and Glut-4 in human hearts as well. Previously, a downregulation of Glut-4 in human hearts with end-stage heart failure has been reported; however, Glut-4 downregulation could be reversed by an LV-assist device [50]. Similarly, at least in the SHRs used here, downregulation of Glut-4 was attenuated by spironolactone.

## 5. Study Limitations

There are two major limitations in this study that could be addressed in future research. First, as we used the diuretic agent spironolactone, we cannot clearly discriminate whether an anti-hypertensive effect or a possible direct effect of the drug occurred. However, the observed effect on blood pressure was limited, as the rats still remained hypertensive. Moreover, the reduced blood pressure was not sufficient to reduce myocardial hypertrophy, but improved EF. Nevertheless, these experiments were performed as a curative treatment after six months of hypertension, aimed to prevent an induction of UCP-2. Therefore, an important finding of this study is that hypertension alone does not induce a nonreversible effect on UCP-2 and Glut-4 expression.

The second is that we still have to elucidate the mechanism of how Glut-4 metabolism is regulated by UCP-2 expression and how UCP-2 expression affects mitochondrial ATP production. For future experiments, it would, therefore, be important to investigate turn-over rates for ATP under UCP-2 silencing in vitro and during compensation and decompensation in hypertrophic hearts in vivo.

## 6. Conclusions

In summary, the results of this study demonstrate for the first time that UCP-2 seems to influence the cardiac glucose metabolism by affecting glucose uptake through Glut-4. We suggest that low UCP-2 levels are pivotal to maintain the adaptive phase of cardiac hypertrophy in the concomitant presence of hypertension, whereas an increased expression of UCP-2 reduces the ability of cardiomyocytes to uptake glucose in a situation where they have lost their metabolic flexibility.

## Supplementary Materials

The following are available online at https://www.mdpi.com/2073-4409/9/3/552/s1, Table S1: Description of all primers used in this study.

## Abbreviations

Ang-II	angiotensin II
ANP	atrial natriuretic peptide
EF	ejection fraction
FCS	fetal calf serum
GAPDH	glyceraldehyde 3-phosphate dehydrogenase
Glut-4	glucose transporter type 4
HepG2	hepatocelluar carcinoma
Hprt	hypoxanthine phosphoribosyl transferase
hPSC	human pluripotent stem cells
hUCP2	human UCP-2 protein
LVW/TL	left ventricular weight normalized to tibia length
MRA	mineralocorticoid receptor antagonist
PBS	phosphate buffered saline
PGC-1α	peroxisome proliferator-activated receptor gamma coactivator 1-alpha
RAAS	renin-angiotensin-aldosterone-system
ROS	reactive oxygen species
SD	standard deviation
SE	standard error
scRNA	scrambled RNA (control)
SHR	spontaneously hypertensive rats
siRNA	small interfering RNA
UCP-2	uncoupling protein 2
