# Supplementary material for: Alterations in Glucose Metabolism During the Transition to Heart Failure: The Contribution of UCP-2
Source: Cells. 2020 Feb 27;9(3):552. doi: 10.3390/cells9030552 (PMC7140436; doi:10.3390/cells9030552)
Supplement: Supplementary file 1 [file cells-09-00552-s001.pdf]

SUPPORTING INFORMATION

Alterations in Glucose Metabolism During the Transition to Heart Failure: The Contribution of UCP-2

Hanna Sarah Kutsche [1*], Rolf Schreckenberg [1], Martin Weber [1], Christine Hirschhäuser [1], Susanne Rohrbach [1], Ling Li [1], Bernd Niemann [2], Rainer Schulz [1], Klaus-Dieter Schlüter [1]

[1] Physiologisches Institut, Justus-Liebig-Universität, 35392 Gießen, Germany
[2] Universitätsklinikum Gießen, Klinik für Herz-, Kinderherz- und Gefäßchirurgie, 35392 Gießen, Germany
* Correspondence: hanna.kutsche@physiologie.med.uni-giessen.de; Tel.: +49 641 99 47145 (H.K.)

Content:

Table S1: Description of all primers used in this study

Table S1. Description of all primers used in this study

**ANP**	Forward	ATG GGC TCC TTC TCC ATC AC
	Reverse	TCT TCG GTA CCG GAA GCT G
	Product length	456 bp
	NCBI reference	NM_012612
**HPRT**	Forward	CCA GCG TCG TGA TTA GTG AT
	Reverse	CAA GTC TTT CAG TCC TGT CC
	Product length	132 bp
	NCBI reference	NM_012583
**PGC-1α**	Forward	AGT GCT CAG CCG AGG ACA CGA
	Reverse	TGC CCC TGC CAG TCA CAG GA
	Product length	180 bp
	NCBI reference	NM_031347
**Slc2a4 (Glut-4)**	Forward	ACC GTC TTC ACG TTG GTC TC
	Reverse	ATC AAG ATG GCA CAG CCA CA
	Product length	101 bp
	NCBI reference	NM_012751.1
**UCP-2**	Forward	CAA TGT TGC CCG AAA TGC CA
	Reverse	TCT TGA CCA CAT CAA CGG GG
	Product length	176 bp
	NCBI reference	NM_019354.3

ANP, atrial natriuretic peptide; HPRT, hypoxanthine phosphoribosyl transferase; PGC-1α, peroxisome proliferator-activated receptor gamma coactivator 1-alpha; Slc2a4, solute carrier family 2 member 4, Glut-4; UCP-2, uncoupling protein-2
